# Aggregation-Induced Emission Luminogens: A New Possibility for Efficient Visualization of RNA in Plants

**DOI:** 10.3390/plants13050743

**Published:** 2024-03-06

**Authors:** Zheng-Chao Yang, Li-Xiang Zhao, Yu-Qi Sang, Xin Huang, Xuan-Chen Lin, Zhi-Ming Yu

**Affiliations:** College of Life and Environmental Sciences, Hangzhou Normal University, Hangzhou 311121, China; zyaire1113@outlook.com (Z.-C.Y.); 2020211506056@stu.hznu.edu.cn (L.-X.Z.); syq20021128@icloud.com (Y.-Q.S.); 19123255187@163.com (X.H.); xc727233459@163.com (X.-C.L.)

**Keywords:** aggregation-induced emission (AIE), RNA fluorescence labeling, click chemistry, RNA aptamer

## Abstract

RNAs play important roles in regulating biological growth and development. Advancements in RNA-imaging techniques are expanding our understanding of their function. Several common RNA-labeling methods in plants have pros and cons. Simultaneously, plants’ spontaneously fluorescent substances interfere with the effectiveness of RNA bioimaging. New technologies need to be introduced into plant RNA luminescence. Aggregation-induced emission luminogens (AIEgens), due to their luminescent properties, tunable molecular size, high fluorescence intensity, good photostability, and low cell toxicity, have been widely applied in the animal and medical fields. The application of this technology in plants is still at an early stage. The development of AIEgens provides more options for RNA labeling. Click chemistry provides ideas for modifying AIEgens into RNA molecules. The CRISPR/Cas13a-mediated targeting system provides a guarantee of precise RNA modification. The liquid–liquid phase separation in plant cells creates conditions for the enrichment and luminescence of AIEgens. The only thing that needs to be looked for is a specific enzyme that uses AIEgens as a substrate and modifies AIEgens onto target RNA via a click chemical reaction. With the development and progress of artificial intelligence and synthetic biology, it may soon be possible to artificially synthesize or discover such an enzyme.

## 1. Introduction

In addition to protein-coding mRNA, non-coding functional RNAs play important roles in gene expression and regulation. As the first non-coding RNA (ncRNA) to be discovered and the most abundant RNA in living organisms, ribosomal RNA (rRNA) with peptidyl transferase activity is a central part of ribosome structure and function, catalyzing peptide bond formation [1]. MicroRNA (abbreviated miRNA) (18–24 nt) can induce its complementary mRNA degradation via the RNA-induced silencing complex (RISC) in which it participates [2,3,4,5]. Furthermore, in some cases, miRNAs can also activate gene expression and increase the level of protein translation [6,7]. Long non-coding RNAs (long ncRNAs) commonly have more than 200 nucleotides and can regulate gene expression at multiple levels [8]. Double-stranded RNA (20–24 bp) can interfere with the expression of its homologous endogenous gene [9].

In *Xenopus oocytes*, *Vg1* mRNA is steadfastly localized at the vegetal cortex [10]. The polarized localization of RNA within the cell means that the distribution of RNA is not uniform [11]. In rice, prolamine mRNAs are concentrated on the endoplasmic reticulum (ER) membrane and form a spherical protein body with prolamine intracisternal granules [12,13,14,15]. It is worth mentioning that messenger RNA (mRNA) and small non-coding RNAs are transported between cells and over long distances via the phloem [16,17,18,19,20]. RNA imaging is believed to be the starting point to present possible moving regulatory mechanisms. To track the trajectory of RNA movement in plants, there are two commonly used methods. One is based on fluorescent proteins [21], and the other is based on RNA aptamers [21]. The RNA-labeling method based on fluorescent proteins is an indirect method. Although the method based on RNA aptamers is direct, it is very inefficient and requires the addition of additional fluorophores. This means that to study the trajectory changes in RNA, new labeling technologies must be found.

Aggregation-caused quenching (ACQ) is a common issue when using conventional fluorescent substances, whether they are in solution or solid state [22]. Aggregation-induced emission luminogens (AIEgens) exhibit a phenomenon in which their fluorescence signal increases as the concentration increases due to the limited vibration of the molecule itself [23].

In this review, a detailed introduction to AIE technology and an outlook on the application of this technology to RNA molecules are provided.

## 2. Current RNA-Labeling Tools

### 2.1. GFP Fused to Targeting RNA

RNA bacteriophage coat protein MS2 can interact with an RNA with a stem–loop structure from its phage genome [24]. By integrating the visualization of green fluorescent protein (GFP) and the RNA-binding function of MS2, MS2-GFP has already been used to label RNA localization [25,26,27,28] and to visualize the intracellular trafficking of mobile *Flowering locus T* (*FT*) mRNAs in living plant cells [28,29]. However, the obvious disadvantage of this method is that the 5′ or 3′ end of the target RNA needs to be connected to the RNA with a stem–loop structure through genetic engineering.

### 2.2. Aptamer-Based RNA Labeling

Due to the success of GFP in labeling molecules, an RNA fluorescence technology was developed that mimics the fluorescence principle of GFP.

**Figure 1:**
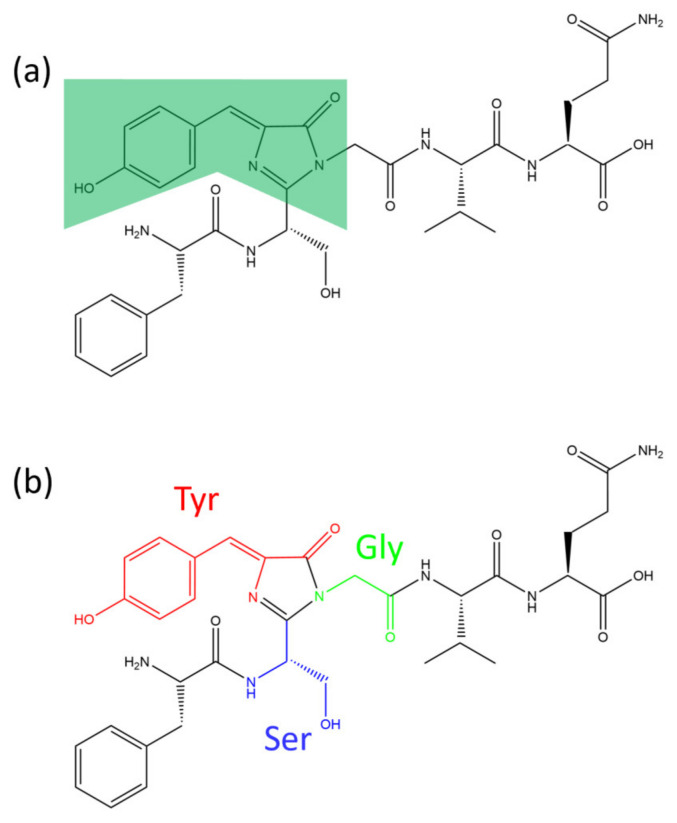
The chromophore of GFP and its photochemistry part. (**a**) The molecular structure marked by green shading is HBI (4-(p-Hydroxybenzylidene)-5-imidazolinone, C_10_H_8_N_2_O_2_) [31]. (**b**) HBI is formed via a cyclization reaction of three amino acids. The red color indicates tyrosine (66th), the blue color indicates serine (65th), and the green color indicates glycine (67th).

Aequorea GFP from *Aequorea victoria* contains a chromophore and amino acids [30]. The chromophore’s molecular 4-(p-hydroxybenzylidene)-5-imidazolone moiety (HBI) is shown in Figure 1 [31].

The chromophore is spontaneously cyclized and oxidized by GFP and three amino acids, Ser65-Tyr66-Gly67, which are protected by GFP [32,33]. Eleven beta-sheet strands and an alpha-helix of GFP form a cylinder [33]. This creates conditions for modification reactions to occur internally. This unique structure has contributed to some internal modifications that can lead to the production of various GFP variants [33].

Based on the luminescence mechanism of GFP and to avoid interference from GFP, Paige et al. [34] started with HBI derivatives to screen fluorescent RNA aptamers using SELEX (systematic evolution of ligands by exponential enrichment) technology in vitro. Fortunately, after multiple rounds of screening and verification at intracellular levels [34], they finally confirmed that 3,5-difluoro-4-hydroxybenzylidene imidazolinone (DFHBI) with 24-2 RNA and 3,5-dimethoxy-4-hydroxybenzylidene imidazolinone (DMHBI) with 13-2 RNA could fluoresce at the cellular level [34].

The combination of DFHBI and 24-2 RNA emits a fluorescent color like that of spinach; thus, it was named Spinach [34]. The photobleaching effect has caused many obstacles to the application of RNA aptamer-based technology in plants, even though there are only two successfully reported cases [35,36]. Even so, multiple RNA aptamer and fluorophore combinations were still developed, as shown in Table 1.

## 3. Aggregation-Induced Emission

Aggregation-induced emission luminogens (AIEgens) have been widely utilized as fluorescent labels in the animal and medical fields [43,44,45,46]. However, their use in plant science has been relatively limited.

### 3.1. ACQ Effect Limits the Application of Traditional Fluorescent Materials

**Figure 2:**
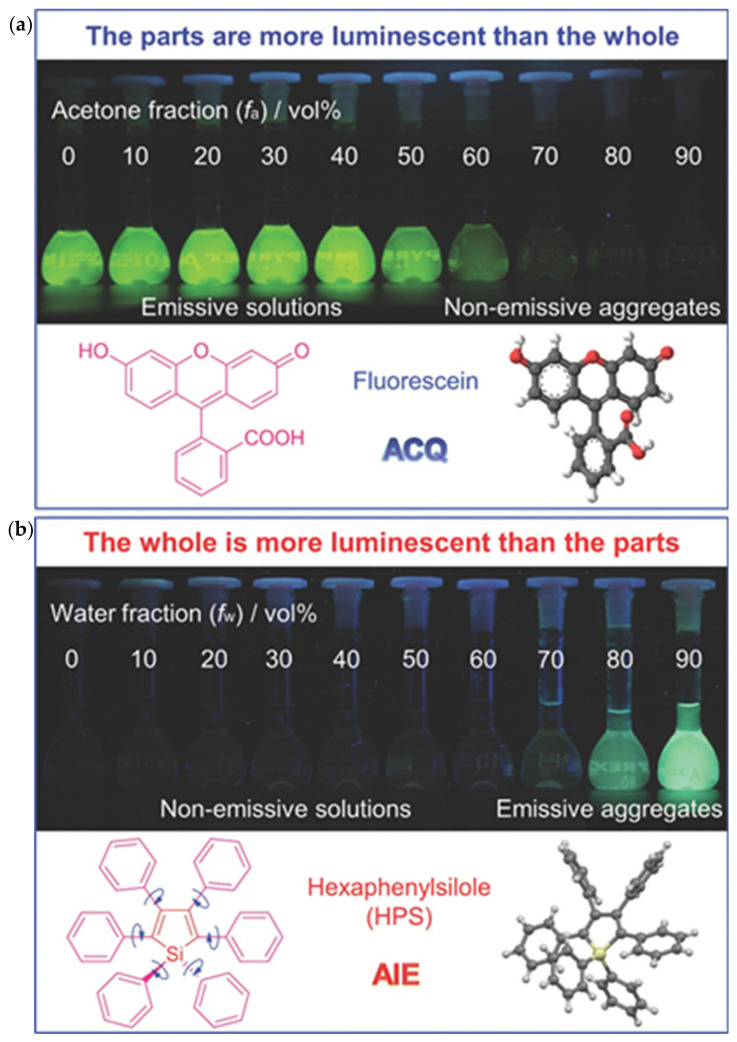
Comparison of ACQ and AIE effects [52]. (**a**) Fluorescein (C_20_H_12_O_5_, 3′,6′-dihydroxyspiro [isobenzofuran-1(3H),9′-[9H] xanthen]-3-one) can be dissolved in water but becomes insoluble in acetone. As the acetone fraction (f_a_) in the acetone aqueous solution gradually increases, the concentration of fluorescein (the initial concentrations are all 15 µM) in the aqueous solution becomes larger and larger. Due to the ACQ (aggregation-caused quenching) effect, the fluorescence of fluorescein becomes weaker and weaker in the aggregated state. (**b**) The solubility property of hexaphenylsilole (C_40_H_30_Si, 1,1,2,3,4,5-hexaphenylsilole, HPS) is opposite to that of fluorescein. HPS has better solubility in organic solvents such as THF (Tetrahydrofuran, (CH_2_)_4_O) rather than water. When the proportion of the water fraction (f_w_) increases, HPS (the initial concentrations are all 20 µM) exhibits increasingly stronger fluorescence due to the so-called AIE (aggregation-induced emission) effect as the aggregation intensity increases. Copyright from reference [52].

Conventional luminescent materials in highly concentrated solutions or solid states will induce the aggregation-caused quenching (ACQ) effect [47,48] (Figure 2). Structurally, conventional fluorescent chromophores are typically rigid planar molecules in large π-conjugated systems composed of planar aromatic rings that emit strong fluorescence when isolated in solution but weaken or even disappear due to molecular aggregation at high concentrations [47,48]. At the molecular level, the stacking of planar aromatic rings increases the degree of π–π coupling between molecules, leading to a significant reduction in luminescence due to energy transfer between low-energy molecules and high-energy molecules via non-radiative leaps [47,48]. ACQ limits the wide application of fluorescent dyes with this property.

### 3.2. The Development of AIEgens

The aggregation-induced emission (AIE) effect was first clearly stated by Luo et al. (2001) [49] (Figure 2). While studying siloles, dropping a 1-methyl-1,2,3,4,5-pentaphenylsilole solution on a thin-layer chromatography plate caused no significant luminescence under UV light. However, the fluorescence enhancement phenomenon was discovered in the dried position of the solution, which led to the discovery of the AIE effect resulting from intramolecular rotation restriction (RIR) (Figure 2). This marked the beginning of a fundamental solution to overcome the ACQ effect [50,51].

Since the initial discovery of AIE in polyphenyl-substituted silicone derivatives, a range of representative compounds such as hexaphenylsilole (HPS) [52], tetraphenylethylene (TPE), and 9,10-stilbenylanthracene (DSA), as well as low-biotoxicity natural AIE effect molecules extracted from plants, such as riboflavin [53], mulberry pigments [54], mangiferin [55], and hematoxylin [56], have been widely developed. Additionally, many AIE-derivative systems have emerged, including aggregation-induced phosphorescence (AIP) [57], crystallization-induced luminescence (CIE) [58], and crystallization-induced phosphorescence (CIP) [59,60].

These compounds provide the possibility for botanists to label target molecules such as RNA.

### 3.3. The Luminescence Mechanism of AIEgens

After more than 20 years, researchers studying the aggregation-induced emission (AIE) mechanism have made some breakthroughs. Some scientists focus on intramolecular action caused by the structure of the molecule itself, while others concentrate on intermolecular action resulting from the interactions of molecules when they are stacked. Six typical mechanisms of AIE luminescence have been identified to date: ① restricted intramolecular motion, ② intramolecular coplanarity, ③ non-compact stacking, ④ formation of J-aggregates, ⑤ inhibition of photophysical processes or photochemical reactions, and ⑥ formation of specific radical conjugates [61].

Tetraphenylethylene (TPE), a typical class of AIE molecules, contains a rotatable benzene ring in its structure, and the energy of its excited state can be consumed through the rotation of the benzene ring in a non-radiative way, resulting in weak fluorescence [62]. However, when the molecule is at a high concentration or in a solid state, the peripheral rotatable aromatic group is limited due to spatial site resistance, and the excited state of the molecule can only decay back to the ground state through the radiation pathway, significantly enhancing luminescence [63]. Additionally, TPE derivatives such as THBDBA and BDBA do not have rotatable benzene rings like TPE but have AIE effects because the energy of their excited state can be consumed through the vibration of benzene rings via non-radiative pathways. Fluorescence is enhanced when intermolecular aggregation prevents this vibration [64]. These two mechanisms are combined as intramolecular rotational restriction (RIR) and intramolecular vibrational restriction (RIV) [63,64].

Despite other AIE mechanisms that complement or act in conjunction with the intramolecular rotational restriction (RIR) mechanism, RIR remains the most mature mechanism based on a new compound.

## 4. Application of AIEgens as Fluorescent Probes and Possibilities for RNA

As a groundbreaking class of organic luminescent materials, AIE compounds have successfully overcome the limitations of traditional fluorescent dyes by avoiding the ACQ effect while retaining their advantages, such as simplicity of operation, high sensitivity, remarkable selectivity, and excellent spatial and temporal resolutions [65]. Due to its significant implications for the human health and agriculture industries, the biological field is gaining more attention, and it is undoubtedly the most promising area for AIE to make a significant impact.

### 4.1. Application of AIEgens in Animal and Medical Fields

#### 4.1.1. Probes for Drug Screening

**Figure 3:**
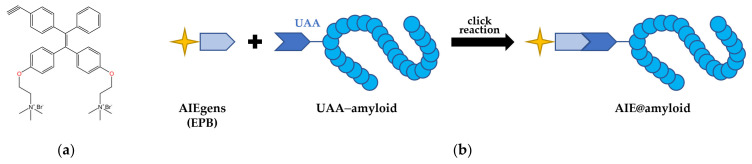
The creation process of AIE@amyloid [70]. (**a**) Chemical structure of EPB (C_38_H_44_Br_2_N_2_O_2_, 2,20-(((2-(4-ethynylphenyl)-2-phenylethene-1,1-diyl) bis-(4,1-phenylene)) bis(oxy)) bis-(trimethylethanaminium) bromide). (**b**) The construction of AIE@amyloid. AIE-active amyloid proteins are synthesized from EPB and UAA–amyloid through a click chemical reaction.

Numerous neurodegenerative diseases, such as Alzheimer’s disease (AD) and Parkinson’s disease (PD), are associated with amyloid self-assembly [66,67]. For instance, β-amyloid (Aβ) is a crucial pathogenic factor in AD that disrupts synaptic plasticity and mediates synaptic toxicity through various mechanisms, whereas PD is linked to the amyloidogenesis of α-synuclein (αSN) [68,69]. To screen general amyloid inhibitors against different amyloid proteins, Jia et al. (2020) designed the AIE-based amyloid inhibitor probe AIE@amyloid [70]. This probe comprises an AIEgen (EPB in this study) and an amyloid protein connected with an unnatural amino acid (UAA) (Figure 3). When amyloid aggregates, the probes also aggregate and emit strong fluorescence. However, the amyloidogenesis of amyloid is prevented in the presence of amyloid inhibitors, resulting in the probes failing to aggregate and the absence of observable fluorescence.

#### 4.1.2. Probes for Micromolecular Biomarkers

Micromolecules, as biomarkers, play important roles in various diseases, making their detection crucial for disease diagnosis [65].

**Figure 4:**
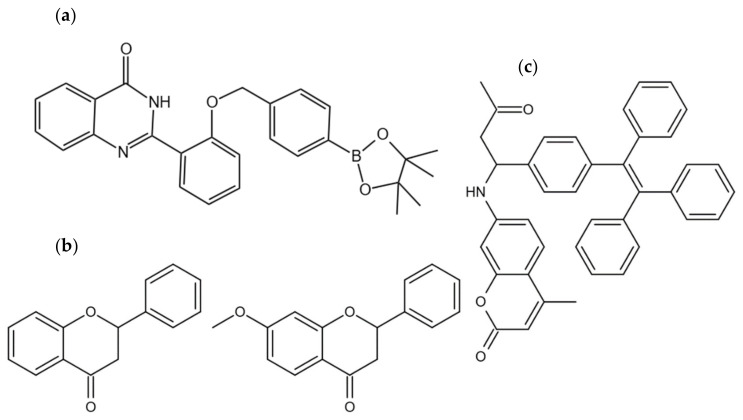
Chemical structures of AIE molecules applied in biological visualization [75,83]. (**a**) The HPQB (C_27_H_26_N_2_BO_4_) structure. (**b**) The structures of flavone (C_15_H_10_O_2_) (**left**) and 7-methoxyflavone (C_16_H_12_O_3_) (**right**). (**c**) Chemical structure of TPE-CA (C_37_H_33_NO_3_).

Hydrogen peroxide (H_2_O_2_) and glucose are two such micromolecules that are closely associated with many diseases and can be used as biomarkers [71,72,73,74]. In a recent study, Wang et al. (2017) developed the AIE-based probe HPQB (C_27_H_26_N_2_BO_4_) [75] for the detection of H_2_O_2_ and glucose (Figure 4a) [76]. The probe is composed of hydroxyphenylquinazolinone (HPQ), which exhibits typical AIE properties, but its fluorescence is quenched by the presence of benzyl boronic pinacol ester. The reaction of H_2_O_2_ with HPQB removes the benzyl boronic pinacol ester, thereby restoring HPQ’s AIE properties and enabling the detection of H_2_O_2_. The study also demonstrated the probe’s ability to image nasopharyngeal carcinoma cells. Reactive oxygen species (ROS), including H_2_O_2_, play an essential role in plant immunity. Thus, the HPQB probe could potentially be used for H_2_O_2_ labeling in plants.

Glutathione (GSH) is another important micromolecular biomarker [77,78]. Xie et al. (2020) developed a GSH-activated probe with a disulfide bond as the GSH response motif [78]. Upon reacting with GSH, the disulfide bond is broken, resulting in the release of the fluorophore and the production of strong fluorescence due to the AIE effect. This probe may be utilized for the detection and imaging of GSH in plants.

#### 4.1.3. Cell Imaging

The visualization of cells or intracellular components provides valuable information on bioprocesses at the cellular or molecular level [79]. Organic fluorescent dyes with excellent optical and biological properties have been used in cell imaging to visualize cells or structures [80]. However, conventional fluorescent dyes are limited by the ACQ effect. AIE compounds, on the other hand, overcome this limitation and have high application value in cell imaging.

Mitochondria, which are the energy centers of cells, produce ATP and support all activities of life. Their dysfunction is associated with various diseases, such as diabetes and cardiovascular disease [81]. Li et al. (2020) selected two AIE compounds (Figure 4b) from a series of flavanones [82]. They found that both compounds showed low cytotoxicity and significant biocompatibility with A549 cells and were specifically aggregated in mitochondria. Their study suggests that these compounds could have applications in imaging mitochondria in living cells, potentially including plant cells.

TPE-CA (synthesized using tetraphenylethene (TPE) and a coumarin (CA) moiety) can specifically display blue fluorescence under the acidic pH conditions of cell organelle lysosomes [83] (Figure 4c).

### 4.2. Application of AIEgens in Plants

The utilization of aggregation-induced emission (AIE) in plants is a nascent field that has garnered significant interest in contemporary times. To date, two major directions have been explored: firstly, the association of AIE with plant photosynthesis to enhance its efficacy, and secondly, the application of fluorescent labeling to a diversity of constituents within plant cells.

#### 4.2.1. AIEgens Enhance the Efficiency of Photosynthesis in Plants

In 2021, Tang’s group utilized click chemistry to incorporate two AIEgens, TPE-PPO and TPA-TPO, into living chloroplasts to enhance photosynthetic efficiency. These AIEgens absorb ultraviolet and green light that is typically unavailable to chloroplasts and convert it into blue and red light, which can be used by chloroplasts [84].

In 2021, using nanotechnology, another group reported that a hybrid photosynthetic system combined chloroplast with CD-AIEgens. It was made of natural quercetin and was able to capture a wider range of light and improve electron transfer efficiency, resulting in enhanced photosynthesis [85].

#### 4.2.2. The Application of AIEgens as Fluorescent Labels in Plant Science

Compared to animal cells, plant cells contain more fluorescent substances that interfere with plant bioimaging. Traditional fluorescent dyes suffer from ACQ, and their usage at high concentrations is limited to avoid fluorescence quenching. Their weak fluorescence at low doses impacts experiments and causes interference when imaging with other fluorescent components like GFP. AIEgens have high stability and luminescence intensity, overcoming the limitations of traditional dyes related to subcellular localization. Various AIEgen-based fluorescent probes have been developed for detecting plant cell substances. In 2017, saponin nanoparticles with AIEgens were used for the first time in Arabidopsis thaliana to fluorescently label plant cell membranes through cell walls, paving the way for AIEgens studies in model plants [86].

Lu et al. (2021) used kaempferol as an AIE fluorescent probe to detect the Al^3+^ concentration in *Arabidopsis thaliana*. The Arabidopsis thaliana roots were incubated in an aqueous solution of kaempferol dissolved in tetrahydrofuran [87]. In addition to the detection of heavy metals in plants, AIEgens can also detect plant hormones such as abscisic acid (ABA). Wu et al. (2022) labeled an AIEgens fluorescent probe to abscisic acid (ABA) in the presence of bovine serum albumin (BSA). This method provided a novel perspective for in vivo or in vitro ABA detection [88].

Although AIEgens have been widely used in the biological field and have great potential for development in botany, AIEgen-related molecular marker technology has seldom been used with plants. Maybe the research on AIEgens labeling RNA has not attracted enough attention. Based on the above application of AIEgens in plants, AIEgens have great potential in RNA-labeling research in plants.

## 5. Outlook

The successful application of fluorescent AIEgens probes in plants indicates the this technology is versatility. As an emerging technology, we give some opinions and suggestions on how AIEgens can be better applied in plant cells.

### 5.1. Click Chemistry for AIE Labeling of RNA

Click chemistry is an efficient method for in vivo biomolecule labeling. If click chemistry is used in combination with AIEgens on RNA, it shows the highest feasibility as a fluorescent probe for RNA in plants. The most used organic reaction in click chemistry is the azide–alkyne cycloaddition, which utilizes compounds modified with alkyne bonds and organic azides for catalysis by Cu [89,90]. Previously, the use of click chemistry in living organisms was greatly limited by the need for a copper catalyst that was toxic to bacteria and mammalian cells. Fortunately, in 2004, the development of the strain-promoted azide–alkyne cycloaddition overcame this limitation and allowed for the performance of click chemistry in living animals without causing physiological damage, demonstrating great potential for non-invasive imaging applications [91].

In 2008, Jao et al. demonstrated the incorporation of a substance with an alkyne bond, such as 5-ethynyluridine (EU), into mRNA via transcription, followed by copper (I)-catalyzed alkyne–azide cycloaddition using fluorescent azides in living organisms [92]. This experiment fully demonstrated the feasibility of fluorescent group modification of biomolecules using click chemistry in living organisms. Another successful case is [Ru(phen)2(4,7-dichloro-phen)]^2+^, which contains a halogen bond that can attach to nucleolar ribosomal RNA and aggregate luminescence for in situ tracing of nucleolar ribosomes [93].

**Figure 5:**
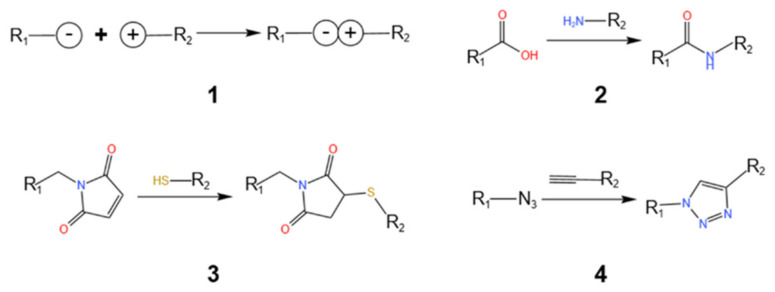
Several click chemistries patterns of AIEgens linked to biological macromolecules. (1) Two groups containing different charges form new compounds through electrostatic interaction. (2) Carboxyl forms carboxamine with compounds containing amino groups. (3) Maleimide reacts with compounds containing sulfhydryl groups to form thioester. (4) Azide forms 1,4-triazole with compounds containing alkynyl groups [94].

There are a few design cases using RNA and AIE together to form fluorescent probes. However, RNA and DNA, as nucleic acid biomolecules, have some structural similarities, and modifications to DNA molecules may be equally applicable to RNA molecules. However, DNA is mostly present in organisms as a double strand. This is different from RNA, which is often present as a single strand. Therefore, more modifications to RNA may be discovered in the future (Figure 5).

### 5.2. For AIE, CRISPR May Be on the Way

The discovery of a highly efficient gene editing system from bacteria has ushered in a new era of molecular biology [95]. The CRISPR/Cas gene editing system is powerful. From the beginning, it only cuts DNA [95,96] to develop DNA [97,98] or RNA [99] base substitutions, cut RNA [100], and label RNA [101]. The development of CRISPR-related technologies is ongoing. So far, there is no perfect report on the real-time imaging of RNA movement trajectories using the CRISPR/Cas system in vivo.

Using RNA-targeting CRISPR/dCas13 (dead Cas13) fused with ADAR2 (adenosine deaminases that act on RNA), adenosine-to-inosine (A-to-I) and cytidine-to-uridine (C-to-U) base exchanges in RNA are realized by ADAR2’s adenine deaminase domain [102,103] and cytidine deaminase domain [99], respectively. This means the CRISPR/Cas13a system provides targeting for functional enzymes, which gives us an idea. Is it possible to fuse a specific click chemistry reaction-performing enzyme to dCas13?

Studies combining click chemistry with RNA targeting have also been reported.

CRISPR/Cas12a, one of a series of CRISPR/Cas systems, combined with click chemistry, was introduced into an electrochemical biosensor for detecting miRNA-21 [103]. This was an amplification reaction for miRNA, but it could not truly reflect the RNA content in the organism.

Liquid–liquid phase separation (LLPS) is an intracellular membrane-less compartmentalization phenomenon that has attracted more and more attention in recent years. It is found in almost all cells of an organism [104].

The site where LLPS occurs is often the place where cells respond to external stimuli. It is also the place where biological macromolecules, especially proteins and RNA, accumulate [104].

**Figure 6:**
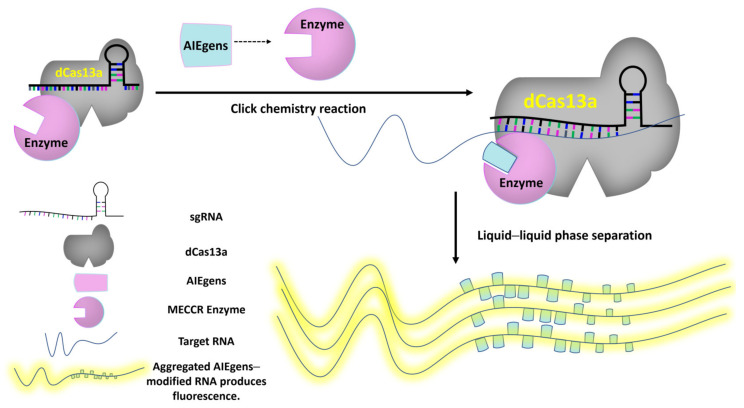
Diagram of AIEgens-modified RNA model based on click chemistry and CRISPR/Cas13a system.

This gives AIEgens an opportunity to fluoresce. Labeling RNA with AIEgens is achieved using enzymes with click chemical reaction characteristics. It is unknown whether these enzymes exist in nature, but with the development of artificial intelligence and synthetic biology, these enzymes are expected to be artificially designed and synthesized (Figure 6).

A modification enzyme with a click chemical reaction function with RNA (MECCR) is fused to the dCas13a protein, which contains sgRNA. When the MECCR enzyme recognizes AIEgens, a click chemical reaction can occur. CRISPR/Cas13a-mediated click chemistry makes AIEgen modification target-specific. When a large amount of target RNA is modified by AIEgens, the RNA molecules generate RNA fluorescence in plant cells based on the aggregation effect of liquid–liquid phase separation.

Among a great variety of nucleic acids editing enzymes, the epigenetic writers of RNA probably have the closest function to MECCR (a modification enzyme with a click chemical reaction function with RNA). Enzymes related to the writers are erasers and readers, which function to remove methylation on RNA and to bind methylated RNA, respectively [105,106,107]. The epigenetic writers are enzymes that methylate RNA [105,106]. The main types of modifications are *N*^6^-methyladenosine (m^6^A), 5-methylcytosine (m^5^C), 5-hydroxymethylcytosine (hm^5^C), *N^1^*-methyladenosine (m^1^A), etc [108]. *N^6^*-methyladenosine (m^6^A) is the most abundant modification in messenger RNA and other types of RNAs and is present in almost all eukaryotes, including humans [108,109], plants [110,111,112], etc.

In *Arabidopsis thaliana*, METTL4 (methyltransferase-like 4, AT1G19340) is one of the methyltransferases (MTase) that specifically catalyzes the N^6^–2′-O-dimethyladenosine (Am) within a single-stranded RNA in vitro [113]. Therefore, we think plant methyltransferase family members are the closest to MECCR. Through retrieval using the IUBMB database, it was found that METLL4 belongs to the EC 2 (enzymes by class of number 2) transferases category [114]. This class of enzymes not only have different functions but also have many members. These advantages provide the possibility for subsequent screening of MECCR.

What is timelier is that the protein structure of AtMETLL4 of *Arabidopsis thaliana* has been solved [113]. In the protein structure, the two tyrosine amino acid sites of Y247 and Y406 play an important role in binding *N^6^* of adenosine; hereafter, the aspartic acid D233 and proline P234 sites play important roles in catalysis and methylation transfer [113]. Since AIEgens molecules are larger than the S-adenosyl-L-methionine (SAM) molecules that provide methyl groups for METLL4, we need to consider when screening and designing MECCR.

## Figures and Tables

**Table 1:** Fluorescence generated by RNA aptamers and paired fluorophores.

RNA Aptamer	Fluorophore	Color	Application	Length of RNA
Spinach [34,36]	DFHBI	Green	HEK-293T, *E. coli*, and onion	98 nt
Spinach2 [37]	DFHBI	Green	*E. coli*, HEK293T, HeLa, and COS-7	95 nt
Pepper [38]	HBC	Green	*E. coli*, 293T/17 HeLa, COS-7, NIH/3T3, U-87, HCT 116, and MKN-45	43 nt
Broccoli [39]	DFHBI-1T	Green	*E. coli* and HEK293T	49 nt
Corn [40]	DFHO	Yellow	*E. coli* and HEK293T	28 nt
3WJ-4 × Bro [35]	DFHBI-1T	Green	*E. coli* and *Nicotiana benthamiana*	1701 nt
Mango [41]	Thiazole Orange (TO1)	Yellow	*E. coli* and *C. elegans*	23 nt
Mango [41]	TO3	Red	*E. coli* and *C. elegans*	23 nt
Mango II [42]	Thiazole Orange (TO1)	Yellow	HEK293T	40 nt

## Data Availability

The data presented in this study are openly available in reference number [31,52,70,75,83,94].
